# Extracranial dose and the risk of radiation-induced malignancy after intracranial stereotactic radiosurgery: is it time to establish a therapeutic reference level?

**DOI:** 10.1007/s00701-020-04664-4

**Published:** 2020-12-15

**Authors:** Ian Paddick, A. Cameron, A. Dimitriadis

**Affiliations:** 1 Queen Square Radiosurgery Centre, National Hospital for Neurology and Neurosurgery, Queen Square, London, WC1N 3BG UK; 2 Bristol Haematology and Oncology Centre, Bristol, BS2 8ED UK

**Keywords:** Radiosurgery, Gamma Knife, Extracranial dose, SRS, Linac, Cyberknife, Radiation-induced malignancy

## Abstract

**Background:**

To measure extracranial doses from Gamma Knife Perfexion (GKP) intracranial stereotactic radiosurgery (SRS) and model the risk of malignancy after SRS for different treatment platforms.

**Methods:**

Doses were measured for 20 patients undergoing SRS on a GKP at distances of 18, 43 and 75 cm from the target, corresponding to the approximate positions of the thyroid, breast and gonads respectively. A literature review was conducted to collect comparative data from other radiosurgery platforms. All data was used to calculate the dose to body organs. The National Cancer Institute (NCI) RadRAT calculator was used to estimate excess lifetime cancer risk from this exposure. Five different age groups covering childhood and younger adults were modelled for both sexes.

**Results:**

Extracranial doses delivered during SRS with the GKP were a median 0.04%, 0.008% and 0.002% of prescription dose at 18 cm, 43 cm and 70 cm from the isocentre respectively. Comparison with the literature revealed that the extracranial dose was lowest from GKP, then linacs equipped with micro-multileaf collimators (mMLC), then linacs equipped with circular collimators (cones), and highest from Cyberknife (CK). Estimated lifetime risks of radiation-induced malignancy in the body for patients treated with SRS aged 5–45 years were 0.03–0.88%, 0.36–11%, 0.61–18% and 2.2–39% for GKP, mMLC, cones and CK respectively.

**Conclusions:**

We have compared typical extracranial doses from different platforms and quantified the lifetime risk of radiation-induced malignancy. The risk varies with platform. This should be taken into account when treating children and young adults with SRS. The concept of a therapeutic reference level (TRL), similar to the diagnostic reference level (DRL) established in radiology, is proposed.

**Supplementary Information:**

The online version contains supplementary material available at 10.1007/s00701-020-04664-4.

## Background

Intracranial stereotactic radiosurgery (SRS) is an established and growing treatment modality for a range of benign diseases including acoustic neuroma, meningioma, pituitary adenoma and arteriovenous malformation (AVM). In addition, a growing number of patients with cerebral metastases are now being treated.

Common contemporary platforms for SRS include the Gamma Knife (GK) (Elekta AB, Stockholm), stereotactic linear accelerators (linacs) such as Novalis (BrainLab GmbH, Germany) and Varian TrueBeam or Edge (Varian Inc., Milpitas, USA), and the Cyberknife (Accuray Inc., Sunnyvale, USA). The design of each platform varies significantly, from beam energy, collimation and beam direction system, image guidance and patient immobilisation systems. This results in variation in leakage and scatter radiation absorbed by the patient’s body. Current published data for the GKP is based on a single treatment of a humanoid phantom, so its range is unknown [18].

Radiation-induced malignancy is widely accepted as a risk of ionising radiation exposures. Though previously controversial, it is now clearly established, through atomic bomb survivors, workers in the nuclear industry, patients receiving radiation therapy and children and adolescents exposed to CT scans, that low dose radiation results in malignancy [3, 5, 10, 20, 25]. Matthews et al. studied the effects of CT scanning in nearly 11 million Australian children and adolescents. 6.2% of the population were exposed to ionising radiation from CT scans estimated at 4.5 mSv per scan. This resulted in 608 excess cancers in the mean 9.5 years of follow-up, equating to 9.4 excess cases of cancer per 100,000 person years at risk [20].

These data are consistent with the Health Protection Agency and International Commission of Radiological Protection’s modelled lifetime risk of cancer after exposure to ionising radiation. The estimates vary with age, sex of patient, dose and area exposed, with the youngest female patients subject to the highest risk [6].

SRS treatment volumes are small resulting in a very low risk of intracranial radiation-induced malignancy which is justified in view of the benefits of disease control [26]. However, the low dose received by the rest of the body, the extracranial dose due to leakage and scatter radiation, will increase the risk of cancer to the whole body. Stereotactic treatments typically require a larger number of monitor units (MUs) compared to conventional radiotherapy treatments. This increases the leakage and scatter radiation from the treatment head and the collimation system resulting in an increase in the whole body dose. This is further exacerbated by increased plan complexity, which typically results in higher numbers of MUs [29].

Once treated, many SRS patients have a normal life expectancy and therefore the late side effects of treatment have the potential to generate a large impact on the quality of survival. This is particularly important for those treated as a child or younger adult, as the patient has many decades to live with the consequences of treatment. The overall risk will depend on the dose the body receives, the age and sex of the patient as well as any inherited susceptibility. While the dose to the body has been shown to vary between different platforms [18], the variation in risk of radiation-induced malignancy has not yet been assessed in the literature.

The aim of this paper is therefore two-fold: firstly, to document the extracranial dose from SRS treatment with the GKP and, secondly, to compare the risk of radiation-induced malignancy after SRS between different treatment platforms.

## Methods

To measure the extracranial dose from the GKP, TMCP Genesis Ultra personal radiations dosimeters, containing four Harshaw LiF:Mg,Cu,P Thermoluminescent Dosimeters (TLDs), from an approved dosimetry service (Mirion Technologies Inc., Berkshire, UK), were used for a series of 20 adult patients. The dosimeters were placed anterior to the patients’ body axis at distances of 18, 43 and 75 cm inferior to their intracranial target, representing the approximate location of the thyroid, breast and gonads. The reported doses were assessed as a percentage of the prescription dose. The effective depth of measurement was 1 cm. Fade characteristics are minimal with this system, and it exhibits a linear range between 1 μGy and 10 Gy. The total uncertainty was 5%.

To compare the extracranial doses from SRS with other stereotactic platforms, a literature review was conducted on PubMed. Publications since 1995 investigating radiosurgery extracranial doses were analysed. Extracranial doses from platforms were plotted as a percentage of prescription dose versus distance from the isocentre, thus eliminating potential bias from different prescription doses between the groups. Where data was recorded as dose to organ, the position of the organ within an average adult was used to convert the measurement point to a distance. Distances within 1 cm were amalgamated. Interpolation was used for comparison where necessary.

For the calculation of extracranial radiation-induced malignancy after SRS, the distance from the isocentre in brain to the organ in the body for an average-sized 5-year-old and average-sized adult was measured for 14 female and 12 male organs. The dose that each organ received from SRS target doses of 12.5 Gy and 25 Gy for four different treatment platforms (GKP, CK, and linacs using cones (cones) and micro-multileaf collimator (mMLC)) was then calculated using the previously plotted extracranial doses. For small organs, e.g. thyroid, the midpoint was taken and a uniform dose was assumed. For longer organs, e.g. lung, a log uniform dose was assumed and the minimum and maximum dose was utilised for calculations.

Lifetime risk of solid radiation-induced malignancy was determined for males and females aged 5, 15, 25, 35 and 45 years at time of exposure from the NCI RadRAT calculator (v. 4.1.1) (for an English population exposed in 2017) for all four treatment platforms [7]. This tool uses lifetime risk models for ten cancers included in the report by the National Academies of Sciences’ BEIR VII Committee plus an additional five cancers developed by the NCI [1]. The calculator utilises Monte Carlo simulation methods with Latin hypercube sampling to estimate the lifetime excess risk. Estimates for three of the additional cancers were excluded: brain, as the estimate was only for extracranial risks; oropharynx, as this organ is very close to the isocentre and the data for the dose to this area was not published; and, ‘other’ as it was not possible to calculate dose to a non-organ. Those aged 15–45 years were assumed to be adult sized.

## Results

Twenty patients had extracranial doses measured during the treatment with GKP of 1–6 intracranial targets: including benign, malignant and functional targets, treated with a median 21 Gy (range 13–80 Gy). The results are summarised in Table 1. The mean/median dose measured at 18, 43 and 70 cm caudal to the intracranial target was 0.044/0.040%, 0.010/0.008% and 0.002/0.002% of the prescription dose respectively.

**Table 1:** A summary of measured extracranial doses for 20 radiosurgery patients treated with Gamma Knife Perfexion

Lesion treated with SRS	Prescription dose (Gy)	Measured dose at 18 cm inf to target in mSv (% of prescription dose)	Measured dose at 43 cm inf to target in mSv (% of prescription dose)	Measured dose at 70 cm inf to target in mSv (% of prescription dose)
Meningioma	15	3.2 (0.02%)	0.9 (0.006%)	0.1 (0.001%)
2 Metastases	22	9.9 (0.05%)	3.4 (0.015%)	0.1 (0.001%)
5 Metastases	22	9.1 (0.04%)	3.0 (0.013%)	0.5 (0.002%)
Vestibular schwannoma	13	9.1 (0.07%)	3.0 (0.023%)	0.5 (0.004%)
6 Metastases	25	5.1 (0.02%)	2.4 (0.009%)	0.3 (0.001%)
2 Metastases	22.25	7.5 (0.03%)	1.9 (0.007%)	0.3 (0.001%)
Pituitary adenoma	24	9.1 (0.04%)	3.0 (0.012%)	0.5 (0.002%)
AVM	18	4.8 (0.03%)	0.9 (0.005%)	0.2 (0.001%)
Meningioma	15	9.1 (0.06%)	3.0 (0.020%)	0.5 (0.003%)
AVM	19	5.4 (0.03%)	1.4 (0.008%)	0.5 (0.003%)
AVM	21	9.6 (0.05%)	1.2 (0.006%)	0.3 (0.001%)
Pituitary adenoma	25	9.7 (0.04%)	1.5 (0.006%)	0.6 (0.002%)
Trigeminal neuralgia	80	5.2 (0.01%)	1.2 (0.002%)	0.3 (0.0004%)
Trigeminal neuralgia	80	6.8 (0.01%)	1.1 (0.001%)	0.2 (0.0002%)
AVM	22	20.2 (0.09%)	2.2 (0.010%)	1.0 (0.004%)
Meningioma	15	8.9 (0.06%)	0.8 (0.005%)	0.3 (0.002%)
Meningioma	15	12.0 (0.08%)	2.5 (0.016%)	0.5 (0.003%)
AVM	25	7.0 (0.03%)	0.8 (0.003%)	0.4 (0.001%)
Vestibular schwannoma	13	9.3 (0.07%)	2.0 (0.015%)	0.4 (0.003%)
Vestibular schwannoma	13	8.2 (0.06%)	1.0 (0.008%)	0.4 (0.003%)
Mean		8.5 (0.044%)	1.8 (0.010%)	0.4 (0.002%)
Median		9.0 (0.040%)	1.7 (0.008%)	0.4 (0.002%)
Min		3.2 (0.006%)	0.8 (0.001%)	0.1 (0.0002%)
Max		20.2 (0.092%)	3.4 (0.023%)	1.0 (0.004%)

A total of four papers were identified as the most comprehensive reports of extracranial doses from modern radiosurgery platforms [9, 13, 18, 29]. Results are recorded in Table 2.

**Table 2:** Extracranial dose in percentage of SRS prescription dose from literature and current series. Numbers in italics are from actual reported distances, numbers in plain text are interpolated from anatomical location to allow comparison between the data

Craniocaudal distance from target (cm)	GKP (this study)	GKP (Lindquist) [18]	Novalis (Gevaert) [13]	Linac mMLC (Di Betta) [9]	Linac Cones (Di Betta) [9]	Cyberknife (Vlachopoulou) [29]	Cyberknife (Di Betta) [9]
Data from:	20 patients	1 humanoid phantom	40 patients	1 humanoid phantom	1 humanoid phantom	21 patients	1 humanoid phantom
15.5				*0.9*	*1.26*		*0.418*
18.0	*0.044*	*0.077*	0.331			2.1	
30.5		*0.035*		*0.20*	*0.36*		*0.182*
34.0			0.189			0.65	
43.0	*0.010*	*0.013*		*0.092*	*0.174*		*0.154*
53.0		*0.0060*		*0.06*	*0.116*		*0.166*
58.5						0.59	
70.0	*0.002*	*0.0027*	0.025				
75.5				*0.026*	*0.052*	0.47	*0.116*
80.5				*0.022*	*0.044*		*0.116*

**Fig. 1:**
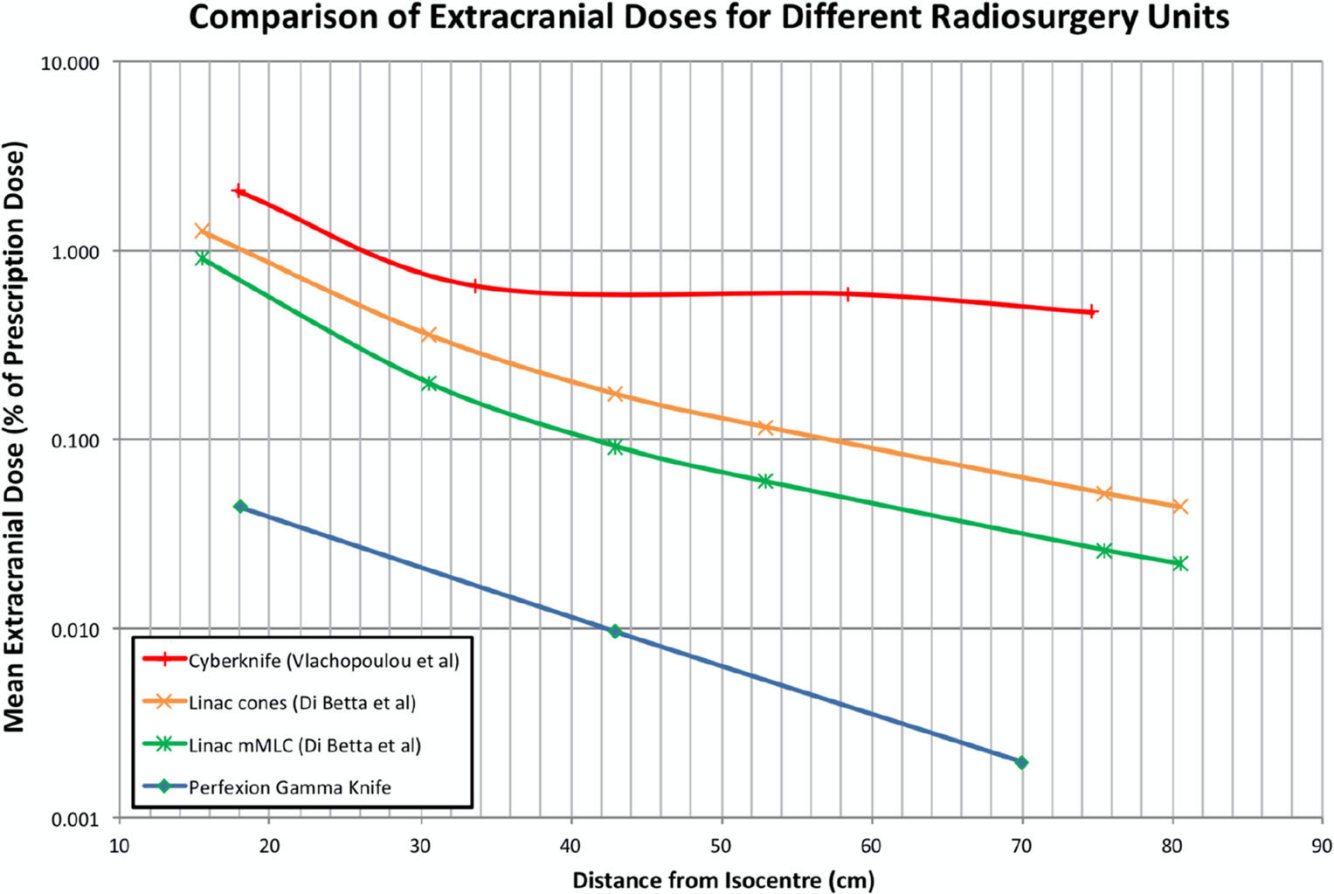
Graph showing extracranial doses from intracranial treatment versus distance from the target for different radiosurgery platforms from the literature

The four platforms used for the calculation of extracranial malignancy risks were Gamma Knife Perfexion, linear accelerator (Philips SL 75–5/Elekta) with mMLC and cones and Cyberknife (post shielding upgrade). The extracranial doses are displayed in Fig. 1.

**Fig. 2:**
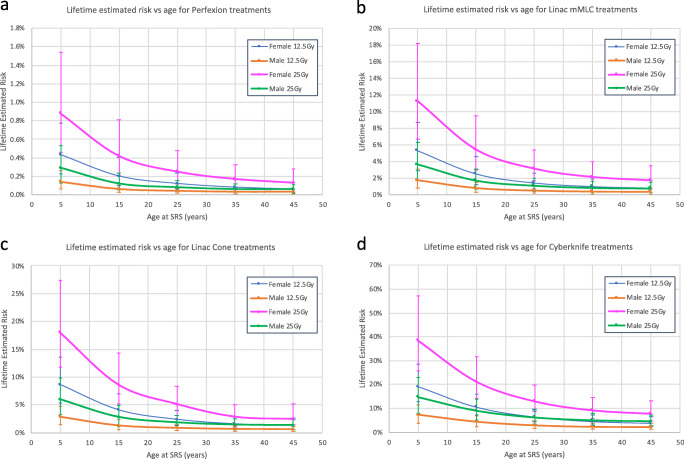
Excess extracranial lifetime risk of cancer treated with intracranial SRS at age 5–45 years old utilising 4 different SRS platforms and 2 different doses in both sexes: **a** Gamma Knife Perfexion. **b** Linac mMLC. **c** Linac cones. **d** Cyberknife

The excess extracranial risk of malignancy varied with age, sex, prescription dose and treatment platform. GKP had the lowest lifetime excess risk: 0.06–0.88% for females and 0.03–0.29% for males aged 5–45 years treated with 12.5–25 Gy SRS. For linac mMLC and cones, the excess risk was 0.78–11% and 1.3–18% (females) and 0.36–3.6% and 0.61–6.0% (males) respectively. For CK, the excess risk was 3.8–39% (females) and 2.2–15% (males) (Fig. 2).

## Discussion

For all patients, exposure to any ionising radiation should be fully justified, acknowledging the risk of radiation-induced malignancy. This includes both the decision to use radiation to treat a disease, when other options (such as surgery) might be possible, and the choice of machine used to treat the patient which will impact on the dose of ionising radiation outside the target. The principles of radiation protection follow the values of ALARA; exposure to radiation should be kept as low as reasonably achievable to minimise this risk.

Intracranial SRS is used to treat a variety of benign diseases. Neurosurgery is often a viable alternative, and in younger patients, where the risk of radiation-induced malignancy is greatest, this may be the treatment of choice. However, there are patients where the risks of surgical resection are significant and the risk benefit ratio favours SRS. Younger patients are often expected to have a life expectancy comparable to their peers without disease, but the exposure to ionising radiation leads them to an increased risk of cancer both within the area of high dose in the brain and the area of low dose extracranially.

Until recently, there was controversy regarding the impact of low dose radiation and its risk for cancer. Some even suggested that it might be beneficial [12]. However, data from substantial studies have suggested that all ionising radiation, however low the dose, increases the risk of malignancy [20, 23, 25]. This linear-no-threshold risk model is the most widely adopted in radiation protection.

In this paper, we have demonstrated that the extracranial dose received during intracranial SRS with the GKP is very low. The mean dose to the thyroid in an adult treated with SRS is 0.044% (range 0.006–0.092%) of the prescription dose and this reduces to 0.002% (range 0.0002–0.004%) in the pelvis. There is a large variation per patient, which is twenty-fold. However, this needs to be viewed alongside the range of volumes treated, which varied by a factor of 50. Larger targets require larger collimators and will therefore have a larger in-patient component of scattered dose. The potential variation of target volumes between each of the platform groups is a limitation when comparing data from the literature, as target volumes were not available. The numbers of patients in each group, which would help to reduce the effect of any outliers, were 20 and 21 for GKP and CK respectively. Our results are very similar to the previously published doses for GKP, while linac and CK doses are also similar to those published in earlier papers [13, 18, 19, 24]. This is likely because all platforms typically treat similar volume targets. Furthermore, Vlachopoulou noted that the effect of collimator size on extracranial dose is limited to areas less than 29 cm from the target, which suggests that target volume (which increases in-patient scatter) may only affect the dose to the head, neck and upper thorax [29].

Although our data does not establish a functional dependence between prescription dose and extracranial dose, the prescription dose is a convenient descriptor facilitating comparison between different SRS treatment platforms, though we acknowledge that this can vary significantly depending on the target volume, shape, isocentre configuration and beam-on time. Our measurements from the 20 cases treated for a variety of conditions improves the robustness of the data. The main weakness of our methodology is that the doses to internal organs are extrapolated from TLD measurements on the surface of the patient’s body rather than internal measurements. This practical limitation of in vivo dosimetry remains a challenge. While measurements in a humanoid phantom could have provided internal organ doses, we opted for external measurements in real patients as we considered that this was more representative of the size and shape of actual patients, which can vary significantly. The prior measurement of extracranial dose in a humanoid phantom treated with the same device gave measurements consistent with ours, which suggests reproducibility between the methods [18].

For a given treatment, organ doses will depend on patient height, as well as patient shape. We wanted to look at the variation in dose for an average adult but used a range of different shaped (and height) patients. This allowed us to collect a range of doses at three fixed distances. Furthermore, the methodology of our study is very similar to the studies that our results are compared with, which enables a more meaningful comparison under the assumption that the difference between surface and organ dose is similar for each platform.

As we have established, the extracranial dose from intracranial SRS varies with a number of factors. One major difference is the platform used. GKP results in an extracranial dose approximately 10 times less than linacs and 100 times less than CK (Fig. 1). There is some variance in the literature for the extracranial dose reported from linac and CK [9, 13, 19, 24, 29]. We chose the Di Betta linac data as it recorded absolute distances from the target. Additional data suggests similar results with other types of linac [13, 19].

Several studies have examined the different components of peripheral dose and the actions that can be taken to reduce this dose [24, 29, 30]. Extracranial dose is a combination of two different sources of extraneous radiation; firstly, radiation scattered from inside the patient, which is proportional to the scattering volume/collimator diameter, and secondly, leakage through, and scatter from, the beam delivery system. The latter will be highly dependent on the number of MUs/treatment time used.

Both of the above elements will also increase with a greater prescription dose. While some of these parameters are patient specific and cannot be altered, some variation in extracranial dose reflects the different planning techniques employed. The treatment planner’s response to target complexity, where intricate targets may be planned with smaller collimators and a larger number of beams to deliver a minimum treatment conformity, will increase the leakage element of dose.

Perhaps of greater importance is the use of non-coplanar beams that can result in more dose being directed towards the body [9, 24, 29]. Beams or arcs entering the vertex of the head will result in the primary beam irradiating and exiting the body. This should be avoided if possible to reduce the body dose. Di Betta et al.’s linac treatments used four non-coplanar arcs and their orientation was so chosen that the exiting beams could not pass through the thyroid. Similar constraints were placed on their CK treatments [9]. Vlachopoulou et al. suggested that their thyroid doses were higher as they did not define the organ as an avoidance structure, though it should be noted that their plans also averaged 2.4 times the number of MUs per Gy compared with Di Betta’s CK plans [29]. In the GKP, it is not possible to direct beams along the length of the body. It is this reason along with the lower energy of the gamma rays (1.25 MeV) and the 18 tons of shielding that the machine is able to employ due to its static beam arrangement that results in this substantially lower extracranial dose.

There are other factors that potentially also affect extracranial dose from intracranial SRS including the choice of MLC or cones, the use of flattening filter free (FFF) treatments with linacs and the use of localisation images.

FFF treatments are being used with increasing frequency for SRS treatments, but there is little definitive data on the extracranial doses received using this method. Kry et al. reported an increase in dose 3 to 15 cm from the field edge when Monte Carlo modelling FFF IMRT delivery [17]. Sharma also found that the whole-body dose was significantly higher when using 6MV FFF beams [27]. However, Cashmore compared paediatric body doses from 6MV FF and FFF IMRT treatments and found that FFF treatments delivered a 64% dose reduction at 50 cm inferior to the target [4]. Therefore, the overall impact of FFF is currently unclear.

While significant but low doses are delivered outside the cranium, these are not necessarily predicted by the planning systems used. Schneider et al. reported that extracranial doses to the patient were underestimated by the CK treatment planning system by a factor of 60, while Huang et al. found an underestimation of 50% of the out of field dose from linac IMRT plans calculated with Pinnacle (Philips, USA) [16]. This is not particularly surprising; dose modelling at distances of many times the beam width from the isocentre is not easy, nor is it normally required in conventional radiation therapy.

For intracranial SRS, there are two methods of ensuring patient position at treatment: fixed frame with no additional imaging or mask with imaging (CBCT or stereoscopic x-rays). This imaging may need to be repeated multiple times to ensure accurate positioning throughout the treatment, and the resulting dose can be significant. Tien et al reported on skin doses from CK treatment for SRS; for those receiving cranial SRS, the average skin dose was 17 cGy (3–53 cGy) [28]. However, the tissue that receives the vast majority of dose from image guidance will be in the treatment region, so, for intracranial radiosurgery, the extracranial dose from this imaging will be relatively low. Doses received from on board imaging will vary, depending on, amongst other factors, field width, mAs, kVp, SSD and frequency of imaging. Imaging will also deliver a much higher skin dose than internal dose. In view of these variable factors combined with the likely low impact on body organ dose, and for the simplicity of this comparison, the extra dose from imaging and therefore the additional risk of malignancy was excluded from our calculations. However, other studies recommend that models should be developed that allow imaging doses to be taken into account [14, 30].

Another factor that we have excluded from our calculations is the impact of fractionation. Though SRS is typically a single dose, some centres opt to treat a similar cohort of patients with hypofractionated protocols where the total dose is often double the SRS dose, while some use conventional fractionation of up to 50–54 Gy in 28–30 fractions, though admittedly with less focussed techniques. How this difference impacts on the risk of radiation-induced malignancy is unclear, though a larger resultant extracranial dose from a larger total prescription dose may result in higher risks. Although fractionation allows repair of sub-lethal damage, the mechanism behind carcinogenesis appears to be different [11]. In terms of our modelling, the NCI RadRAT tool does not take fractionation into account, only whether the exposure is chronic or acute. For this modelling, we used acute exposure. Estimates regarding the lifetime risks of secondary tumours from low doses of radiation are based upon exposures such as the survivors of the atomic bomb, CT scan exposure and nuclear workers, so there is unfortunately no good data to differentiate between low dose exposures from single and multiple fraction radiotherapy.

During the course of this study, the Gamma Knife Perfexion has been superseded by the Gamma Knife Icon. This uses an identical arrangement of sources and collimators, but external elements of the platform have been modified to enable the option of mask-based immobilisation combined with cone beam CT image guidance. This ability of image-guided hypofractionation means that the same uncertainties with regard to risks from imaging and increased doses from fractionation now apply to all platforms investigated in this study.

To establish the risk of radiation-induced malignancy, within the body from intracranial SRS, we used the NCI RadRAT calculator. This calculator is most appropriate for risk estimation for exposures from gamma rays and high energy photons at low doses (< 1 Gy) for a similar group of patients [7]. The calculator, using the same data from the BEIR report, utilises data from the Japanese atomic bomb survivors, with the addition of data from other studies breast and thyroid cancer risks. It assumes that there is no threshold with a linear reduction in risk below 100 mGy. Risk estimate calculations are subject to several sources of uncertainty due to limitations in epidemiological data and in our understanding of exactly how radiation exposure increases the risk of radiation-induced cancer [8]. However, these sources of uncertainty will be similar across the different platforms, so the relative risk provides a more robust assessment (supplementary Table 1). In addition, we excluded risk from irradiation of the oral cavity/pharynx as the doses in the literature were not recorded so close to the isocentre. Irradiation of the bone marrow was also excluded in view of the difficulty estimating the dose to such a disparate organ. Therefore, the risks of radiation-induced malignancy are likely to be a little higher than those estimated but this applies to all platforms.

This paper demonstrates that the risk of radiation-induced malignancy within the body is dependent on the age and sex of the patient, as well as the SRS platform used to provide treatment. Acceptable levels of increased risk have been published by the UK government. The Gamma Knife Perfexion results in an increase in lifetime risk which is low apart from female children [15]. SRS treatment using linac and particularly CK appears to have a substantially higher excess risk, especially in women and even for those at 45 years of age. The relative risk of cancer in the body is 12–14 times higher in those treated with linac compared to Gamma Knife Perfexion and 44–79 times higher in those treated with CK. Though the absolute risk reduces with age, the relative risk does not.

While the dose to the brain is much higher than the body during intracranial SRS, the brain is less prone to secondary tumour induction. This may explain why Rowe et al. did not identify an increase in malignancy in their cohort of 5000 patients treated with SRS [26]. The predicted incidence of malignancy was 2.47 on an age/sex-matched basis, but only one case was observed. However, if sufficient dose is given amongst a large enough population, the excess risks can be seen. A review of SEER data demonstrated a 5 per 1000 excess risk in patients treated with radiotherapy within 15 years after diagnosis [2]. Many more decades of follow-up will be required to observe the actual lifetime risk of malignancy following SRS. For a 5-year-old paediatric patient treated for benign indications such as AVM, life expectancy may be up to 80 years post SRS. In this extreme example, the lifetime excess risk cannot be expected to be observed using current data.

It is the duty of the clinician to ensure that the patient is exposed to the lowest dose of ionising radiation possible. Within the UK, the National Health Service (NHS) has taken the risk of radiation-induced cancer as a high priority. The NHS currently approves the funding of proton treatment overseas for a range of malignant conditions, which have a life expectancy of at least 40% at 5 years, on the basis of reduced late side effects at considerable cost to both the NHS and the patient [21]. A major late side effect that is hoped to be avoided through this policy is the rate of radiation-induced malignancy. Similarly, the risk of malignancy post intracranial SRS has also been taken into account by commissioners of healthcare with the UK SRS commissioning recommending that treatment for those < 25 years is delivered with the lowest possible extracranial dose [22].

In radiology, the concept of the diagnostic reference level (DRL) is routinely employed allowing, for example, comparison of CT scanner doses from the same institution, or at other institutions. This has highlighted the substantial variations in practice between different CT scanners in providing similar examinations. These observations have indicated the need for improvement and the implementation of methods to bring higher doses down to an acceptable range for each investigation. DRLs can provide the stimulus to reduce doses when they are found to be excessive. The Ionising Radiation (Medical Exposure) Regulations 2017 (IR(ME)R 2017) require employers to establish DRLs and to undertake appropriate reviews if they are consistently exceeded [8]. Local DRLs higher than those set nationally would need to be justified.

Similar to the DRL in radiology, we propose the therapeutic reference level (TRL), allowing comparisons between different radiosurgery platforms and encouraging a reduction of extraneous dose in this primarily non-cancer population. This would encourage manufacturers to work on reducing extracranial dose as well as raising the profile of this issue within radiation oncology and neurosurgery departments and thus enabling patients and commissioners to make informed choices. Our suggested value for the TRL in intracranial radiosurgery is 0.01% of the prescription dose at a distance of 60 cm. This value will ensure that patients are protected against needless risks of radiation-induced malignancy in later life. From our cohort of 20 patients, the maximum interpolated dose at this distance with the GKP was 0.004% of the prescription dose, demonstrating safe adherence to this limit.

## Conclusions

Until now, limited extracranial dose data has been available for the GKP. This study increases this knowledge by giving a range of doses likely to be given to patients undergoing radiosurgery with this device.

Extracranial doses received by patients undergoing radiosurgery are a source of radiation exposure, often to non-cancer patients. For the treatment of benign disease with radiation, risks of malignant induction must be evaluated and patients should understand the risks involved. This needs to be weighed up with the risks of alternative techniques such as microsurgery.

These risks vary dramatically depending on the age and sex of the patient, the radiosurgery platform used, and therefore should be considered when offering treatment.

## Supplementary Information

ESM 1 (DOCX 13 kb)
