# Supplementary material for: Extracranial dose and the risk of radiation-induced malignancy after intracranial stereotactic radiosurgery: is it time to establish a therapeutic reference level?
Source: Acta Neurochir (Wien). 2020 Dec 15;163(4):971–9. doi: 10.1007/s00701-020-04664-4 (PMC7966618; doi:10.1007/s00701-020-04664-4)
Supplement: Supplementary file 1 — (DOCX 13 kb) [file 701_2020_4664_MOESM1_ESM.docx]

Supplemental Table 1. Percentage relative risk of excess extracranial cancer after intracranial SRS at different ages and sexes comparing different treatment platforms

| Relative Risk (%) | GKP v mMLC 12.5 Gy | GKP v mMLC 25 Gy | GKP v cones 12.5 Gy | GKP v cones 25 Gy | GKP v CK 12.5 Gy | GKP v CK 25 Gy | mMLC v CK 12.5 Gy | mMLC v CK 25 Gy |
| --- | --- | --- | --- | --- | --- | --- | --- | --- |
| Female 5 Year Old | 12.2 | 12.7 | 19.9 | 20.4 | 44.2 | 43.7 | 3.4 | 3.6 |
| Female 15 year old | 12.3 | 12.8 | 19.9 | 20.5 | 51.2 | 50.6 | 4.2 | 4.0 |
| Female 25 year old | 11.9 | 12.6 | 19.8 | 20.6 | 52.6 | 52.4 | 4.4 | 4.2 |
| Female 35 year old | 11.9 | 12.6 | 19.9 | 20.7 | 55.4 | 55.4 | 4.7 | 4.4 |
| Female 45 year old | 12.1 | 12.9 | 20.3 | 21.1 | 59.1 | 59.1 | 4.9 | 4.6 |
| Male 5 year old | 12.3 | 12.7 | 20.4 | 20.9 | 51.7 | 51.8 | 4.2 | 4.1 |
| Male 15 year old | 13.5 | 14.0 | 22.2 | 22.6 | 73.8 | 74.4 | 5.5 | 5.3 |
| Male 25 year old | 13.3 | 13.8 | 22.1 | 22.6 | 75.5 | 76.7 | 5.7 | 5.5 |
| Male 35 year old | 13.0 | 13.8 | 21.9 | 22.7 | 77.0 | 79.1 | 5.9 | 5.8 |
| Male 45 year old | 13.0 | 13.6 | 22.0 | 22.6 | 78.2 | 79.4 | 6.0 | 5.8 |
